# Deciphering the sensing of α-amyrin acetate with hs-DNA: a multipronged biological probe†

**DOI:** 10.1039/d1ra07195e

**Published:** 2022-01-05

**Authors:** Amol V. Pansare, Amol A. Shedge, Maryappa C. Sonawale, Shubham V. Pansare, Akshay D. Mahakal, Shyam R. Khairkar, Shraddha Y. Chhatre, Dnyaneshwar K. Kulal, Vishwanath R. Patil

**Affiliations:** Composite Group, Swiss Federal Laboratories for Materials Science and Technology-Empa 8600 Dübendorf Switzerland amol.pansare@empa.ch; Department of Chemistry, University of Mumbai Santacruz (E) Mumbai 400098 India vishwanathrpatil03@gmail.com; Veer Wajekar Arts Science and Commerce College Navi Mumbai 400702 India; National Chemical Laboratory (NCL) Dr. Homi Bhabha Road Pune 411008 India

## Abstract

In this study, we focus on the biomimetic development of small molecules and their biological sensing with DNA. The binding of herring sperm deoxyribonucleic acid (hs-DNA) with naturally occurring bioactive small molecule α-amyrin acetate (α-AA), a biomimetic – isolated from the leaves of *Ficus* (*F.*) *arnottiana* is investigated. Collective information from various imaging, spectroscopic and biophysical experiments provides evidence that α-AA is a minor groove sensor of hs-DNA and preferentially binds to the A–T-rich regions. Interactions of different concentrations of small molecule α-AA with hsDNA were evaluated *via* various analytical techniques such as UV-Vis, circular dichroism (CD) and fluorescence emission spectroscopy. Fluorescence emission spectroscopy results suggest that α-AA decreases the emission level of hsDNA. DNA minor groove sensor Hoechst 33258 and intercalative sensor EB, melting transition analysis (*T*_M_) and viscosity analysis clarified that α-AA binds to hs-DNA *via* a groove site. Biophysical chemistry and molecular docking studies show that hydrophobic interactions play a major role in this binding. The present research deals with a natural product biosynthesis-linked chemical–biology interface sensor as a biological probe for α-AA: hs-DNA.

## Abstract 2

In this study, we focus on the biomimetic development of small molecules and their biological sensing with DNA.

## Graphical abstract

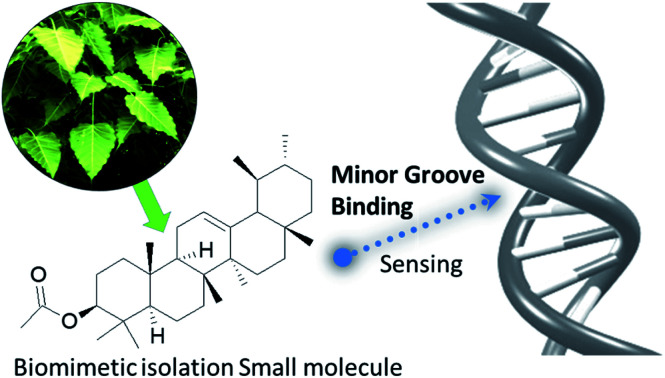

## Introduction

Drugs for clinical use require careful investigation and design to evaluate their binding interaction with DNA, and hence, their toxicity.^1,2^ Binding modes and binding interactions of molecules with DNA provide vital data to understand the mechanism of action of drug molecules. α-Amyrin acetate (α-AA), a bioactive triterpenoid isolated from the leaves of *Ficus* (*F.*) *arnottiana*, shows antihyperglycemic activity and improved atherogenic lipid profiles in rats, suggesting that α-AA can be used as an effective antidiabetic cum lipid-lowering agent for type 2 diabetes mellitus.^3–5^ In another study, it showed anti-inflammatory activity and aphrodisiac properties in male rats. α-AA also prevented the increased serum aspartate aminotransferase and serum alanine aminotransferase levels during inflammation.^5^

A literature survey revealed that α-AA exhibited antihyperglycemic activity and anti-inflammatory activity.^6^ α-AA also acts as tyrosinase inhibitor,^7^ hepatoprotective active agent,^8^ it can be used as a natural source of insect growth regulator and monoamine oxidase inhibitory agent.^9,10^ α-AA has also been isolated from *Tabernaemontana dichotoma* and tested for its antidiabetic potential. α-AA was one of the major constituents isolated from the benzene extract of *Alstonia scholaris* bark and showed antifertility activity in male rats.^11^

Increasing importance of α-AA triterpenoid, hence numerous researchers have isolated the bioactive molecule from various plant species *viz. Calotropis gigantean* (L.) Dryand (Asclepiadaceae) family, leaves of *Hoya paziae* Kloppenb, and *Tabernaemontana stapfiana* Britten.^12–14^ β-AA and α-AA isolated from *Abroma augusta* L., *Dorstenia arifolia* and *Alstonia boonei* exhibited anti-inflammatory activity.^15–17^ α-Amyrin and β-amyrin from *Protium heptaphyllum* exhibited antihyperglycemic and hypolipidemic effects in mice. α,β-Amyrin from *Telfairia occidentalis* Hook F. (Cucurbitaceae) exhibited prominent anti-oxidant activity.^18,19^ α-Amyrin from *Rhaponticum carthamoides* induced the proliferation of human keratinocytes (HaCaT) promoting wound healing and skin regeneration.^20^

The efficiency and efficacy of a drug molecule can be directly related to its DNA binding.^21,22^ Studying the interaction of DNA is of prime importance in drug design. For example Metformin is the most commonly prescribed oral antihyperglycemic drug in the world. However, recently, it has been shown to induce DNA damage within mammalian cells.^23^ The study also indicated that chronic metformin exposure could be potentially genotoxic.

Furthermore, the interaction of DNA with Metformin–Ru(ii)–arene and palladium complexes *via* absorption and emission techniques have recently suggested hydrophobic bonding between Metformin and DNA.^24,25^ Another point of concern for potent drug molecules interacting with DNA is their ability to cause secondary cancers after elimination of the primary disease.^26^

**Fig. 1:**
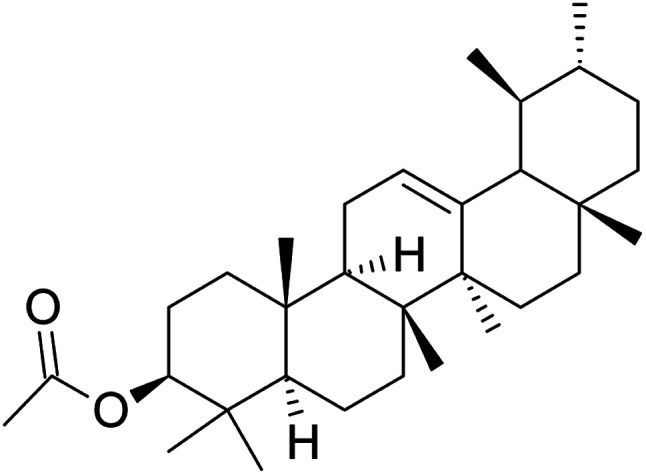
Structure of α-amyrin acetate.

Despite its bioactivity, no attempt has been made to study the interaction between α-AA (Fig. 1) and DNA. Using a comprehensive multi-spectrometric approach and molecular docking studies, we determined the binding affinity and binding thermodynamics specific to the interactions of α-AA with hs-DNA. Investigations from UV-visible spectroscopy, fluorescence, melting temperature (*T*_M_), hydrodynamic experiments and molecular docking studies provided significant information about the nature of binding of α-AA to hs-DNA.

## Experimental

### Materials

Ethidium bromide (EB), hs-DNA, sodium phosphate and Hoechst 33258 were purchased from Sigma-Aldrich, India.

### Methods

The detailed binding behavior of α-AA with the hs-DNA molecule was analyzed meticulously. Interaction between α-AA and DNA was determined by UV-Vis absorption, fluorescence measurements, CD measurements, viscosity experiments; thermal denaturation and minor groove displacement assays were demonstrated by a previously reported method.^27^ Glide version 5.7 – Schrödinger suite 2013-1 was used for the molecular docking of α-AA–DNA binding. The B-DNA dodecamer d(CGCGAATTCGCG)_2_ (PDB ID: 1BNA) structure was copied from the protein data bank (http://www.rcsb.org./pdb).

α-AA was isolated from *F. arnottiana*, and the detailed procedure is summarized in the ESI† with detailed characterization – FTIR and NMR data (Fig. S2–S4†).

The hs-DNA concentration was evaluated *via* absorption spectroscopy (*λ* = 260 nm) molar extinction coefficient (13 200 M^−1^ cm^−1^).^2^ A stock solution of α-AA (1.0 × 10^−3^ mol L^−1^) in 5% ethanolic buffer was utilised. Further dilutions were made using only the buffer solution. A stock solution (2.0 × 10^−3^ mol L^−1^) of EB was prepared by dissolving in a Tris–HCl buffer solution. UV-Vis absorption spectra of hs-DNA were recorded on a LAB UV3000^plus^ spectrophotometer in the absence and presence of α-AA at 298 K in the range of 200–600 nm.

### Absorption study

**Fig. 2:**
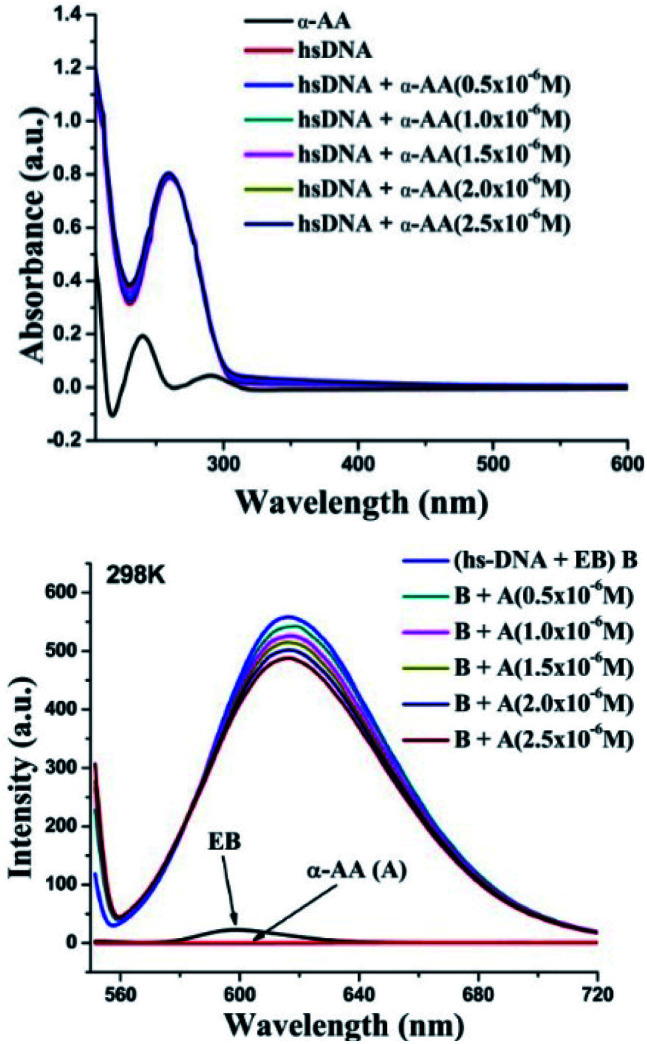
(Top) Interaction of α-AA (0.25 × 10^−6^ M) with hs-DNA (50 × 10^−6^ M) using UV-Vis spectroscopy. UV-absorption spectra of hs-DNA in presence of various α-AA concentrations in 0.5 M Tris–HCl buffer (pH = 7.2). (Ratio: 0.5–2.5/50) [α-AA]/[DNA]. (Down) Fluorescence titration of the EB–hs-DNA (B) complex with α-AA. EB–hs-DNA complex emission intensity were recorded from 550–720 nm and excited at 526 nm.

α-AA has a characteristic, well-defined intense peak at 240 nm, but has a very weak absorption coefficient compared to hs-DNA. (*ε* (α-AA) = 234 M^−1^ cm^−1^, *ε* (hs-DNA) = 13 200 M^−1^ cm^−1^),^2^ as shown in Fig. 2. hs-DNA has an absorption peak at 260 nm and the absorption remains practically constant upon increasing the concentration of the α-AA solution without any blank interference (Fig. S4 and S5†). Hyperchromism is a phenomenon of increase in the absorption intensity, which originates from the breaking of the secondary structure of DNA, while hypochromism results from the stabilization of the helical structure of DNA, either by intercalation or by electrostatic interaction.^28^

No hyperchromism or hypochromism is observed on the successive addition of α-AA, indicating that the helical structure of hs-DNA is conserved. Hence, a groove binding mechanism might be more plausible.^29^

Phosphate groups of DNA are often responsible for electrostatic interactions with small molecules. UV-Vis spectroscopy was used to study the binding possibility of phosphate groups to α-AA.^30^ Increasing concentration of phosphate groups (originating from the buffer) were added to α-AA (Fig. S6†). There was negligible change during the titration, ruling out electrostatic interactions between α-AA and the phosphate groups of DNA.

### Fluorescence quenching

Probing the interaction with fluorescence quenching: hs-DNA itself has almost no fluorescence. Therefore, ethidium bromide (EB), a mutagenic intercalating dye was used as a fluorescence probe to investigate the interaction of α-AA with hs-DNA.^31,32^ It is known that the planar aromatic rings of ethidium bromide intercalate to the base pairs of DNA.^33,34^ The intrinsic fluorescence of EB is low (Fig. 2). However, on the addition of EB, the fluorescence intensity of hs-DNA was significantly increased, as reported previously^33,34^ Upon complex formation, the hs-DNA–EB complex exhibited an emission maximum at 617 nm (excitation at 526 nm). On titrating a constant concentration of the hs-DNA–EB complex with increasing concentrations of α-AA, the quenching of the fluorescence intensity of hs-DNA–EB was observed (Fig. 2) without any noticeable shift in the emission maximum. This might be due to three possible reasons:^35,36^

**Fig. 3:**
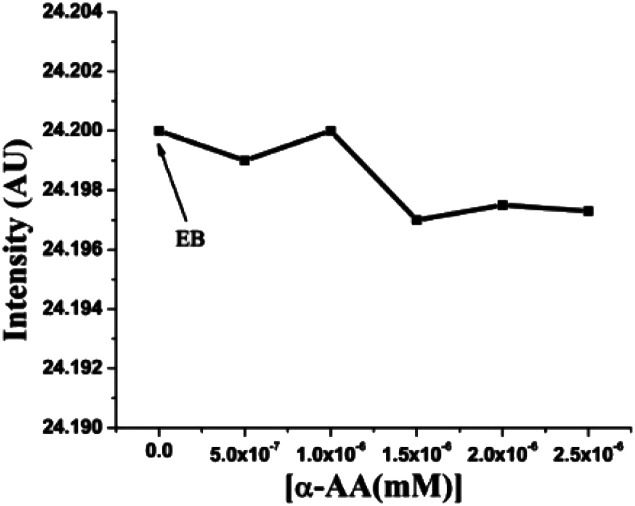
Fluorescence of EB (concentration 4 × 10^−6^ M) with distinct concentrations of α-AA (0.5–2.5 × 10^−6^ M).

First, the interaction of α-AA with EB can cause fluorescence quenching. However, we found that the addition of α-AA did not cause a significant change in the fluorescence of EB (Fig. 3), indicating that α-AA does not interact with EB.

Second, α-AA may displace EB from the DNA–EB complex, which would result in a decrease in the concentration of the EB–DNA complex. The known binding constant *K* value of DNA–EB is 5.16 × 10^5^ mol L^−1^,^37^ and the resultant binding constant *K* value of α-AA and DNA–EB was 3.522 × 10^4^ mol L^−1^, (see ESI, Table S1†) suggesting that replacement of EB from hs-DNA was improbable. The third possibility is that a new complex α-AA–hs-DNA–EB is formed *via* groove binding.^38^

**Fig. 4:**
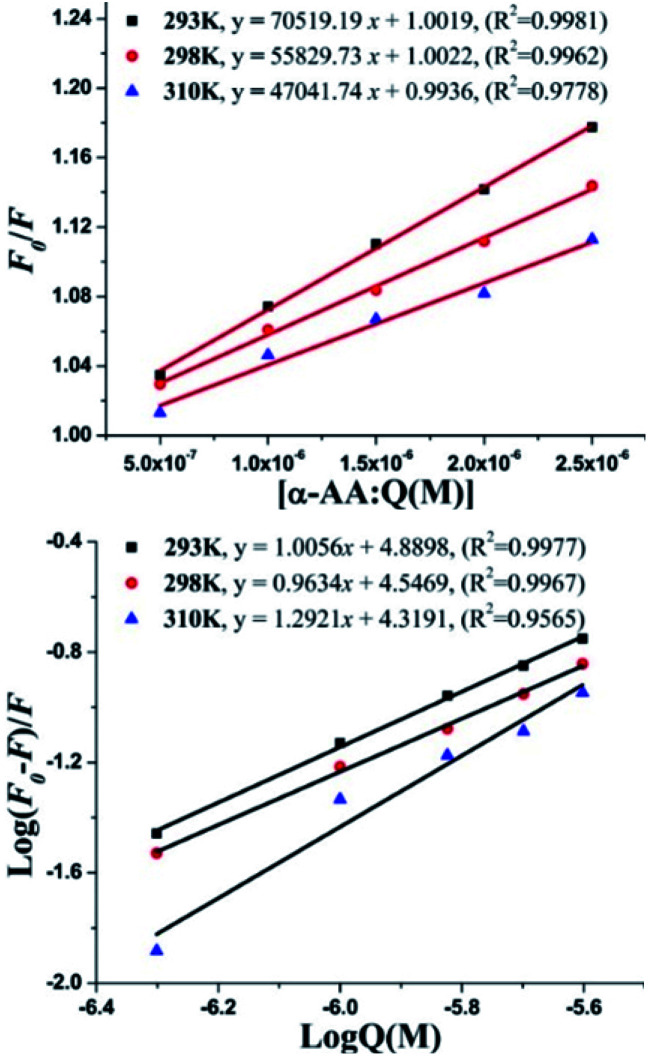
(Top) Stern–Volmer plot for the fluorescence quenching of hs-DNA with α-AA in Tris–HCl buffer at three different temperatures. (Down) Modified Stern–Volmer plot of Log(*F*_0_ − *F*)/*F versus* log[*Q*] at three different temperatures. [*Q*] = concentration of α-AA.

Fluorescence quenching can occur *via* two mechanisms – (i) dynamic quenching, which involves collisional encounters between the fluorophore and the quencher (increase in the diffusion coefficient with increase in temperature) and (ii) static quenching, which is due to a decrease in the stability of the complex (decreased quenching constant (*K*_q_) with an increase in temperature). To investigate the fluorescence quenching mechanism of the interaction between α-AA and hs-DNA, we performed fluorescence spectroscopy experiments at three temperatures (293 K, 298 K and 310 K). From the Stern–Volmer plot Fig. 4 and eqn (S1),†*F*_0_/*F vs.* concentration of α-AA at three different temperatures, we obtained the binding constant (*K*_sv_) and the quenching constant (*K*_q_) (Table S1†).^39,40^ Quenching constant values were distinctly greater compared to the value of scattering collision quenching constant (2.0 × 10^10^ L mol^−1^), suggesting a static quenching mechanism.^41^ The number of binding sites (*n*) and binding constant (*K*) between α-AA and hs-DNA was obtained from the plot of log[(*F*_0_ − *F*)/*F*] *vs.* log[*Q*], as shown in Fig. 4, using the modified Stern–Volmer equation (eqn (S2)†).^42^ The values of *K* and *n* are summarized in Table S1.† The *K* values show an inverse proportionality with temperature while *n* ≈ 1. These results suggest the formation of a 1 : 1 complex between α-AA and hs-DNA.

Basically, small molecules bind to the biopolymer through various interactions.^43^ The change in entropy (Δ*S*^0^) and enthalpy (Δ*H*^0^) were obtained from the Vant Hoff's equation (eqn (S3)†).^44^ The Gibbs free energy (Δ*G*^0^) was calculated using eqn (S4).†^45^ From the plot of ln *K vs.* 1/*T* (intercept and slope), Δ*S*^0^ and Δ*H*^0^ were derived accordingly. The values of (Δ*G*^0^), (Δ*S*^0^) and (Δ*H*^0^) are reported in Table S2.† The positive values of Δ*S*^0^ (0.533 kJ mol^−1^ K^−1^) and Δ*H*^0^ (130.48 kJ mol^−1^) indicate that binding occurs *via* hydrophobic interactions.^46^ Negative values of Δ*G*^0^ suggest that the binding between α-AA and hs-DNA occurs spontaneously.

### DNA melting studies

**Fig. 5:**
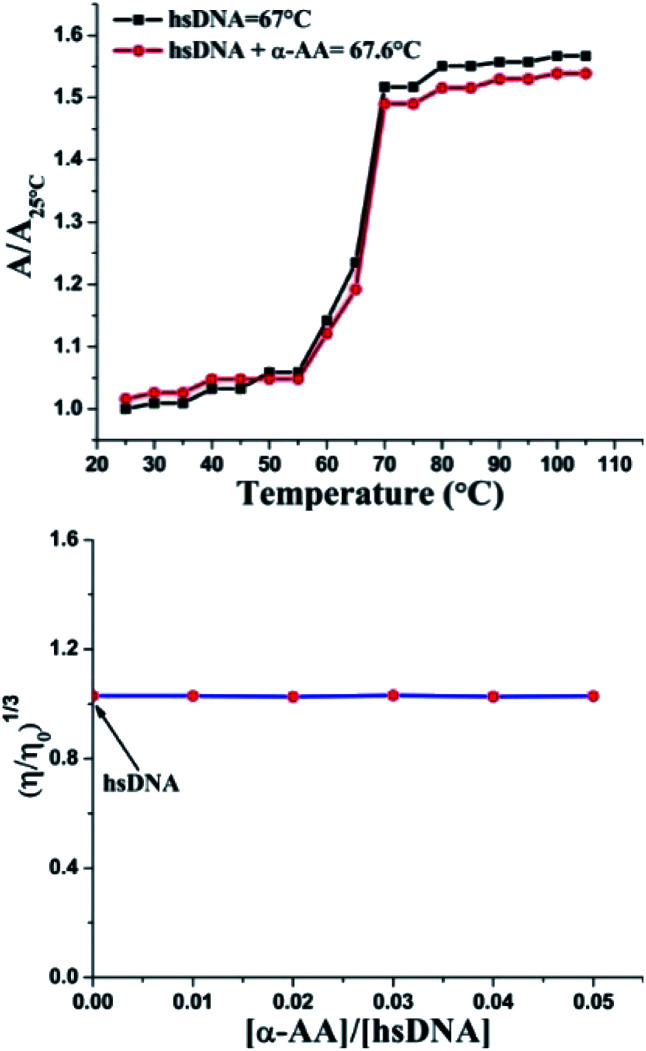
(Top) Melting temperature values of hs-DNA and its complex with α-AA, where specific UV-Vis absorbance illustrated the ratio of hs-DNA within a temperature range of 25–105 °C. Absorbance was monitored at 260 nm. (Down) Effect of increasing amounts of α-AA on the relative viscosity of hs-DNA. Concentration of α-AA was sequentially increased while maintaining a constant concentration of hs-DNA.

An increase in the melting temperature of DNA (usually 5–8 °C) is seen upon intercalative binding to small molecules.^47^ In contrast, no or a slight increase in the melting temperature is observed on groove binding or electrostatic binding.^48^ The melting temperature for hs-DNA was determined from the plot of *A*/*A*_25 °C_ (*A*_25 °C_ is the absorbance at 25 °C and *A* is the absorbance at increasing temperatures, both at 260 nm) *versus* temperature (Fig. 5). The melting temperature of hs-DNA was 67.0 ± 0.1 °C,^49^ while in the presence of α-AA, it was 67.6 ± 1.0 °C. These results indicate the absence of any intercalative mode of binding (Fig. S7†), evident by a negligible change in the melting temperature.

### Investigation of the binding mode

Double helix DNA length is very sensitive against viscosity. Hence, viscosity characteristics have minimal ambiguity and the ultimate reproving test for deciphering the system of binding of miniature molecules to DNA.^50^ Classical intercalators proliferate viscosity of DNA significantly, causing the separation of base pairs at the intercalation sites and leading to an overall increase in the length of DNA.^51^ In contrast, minimal changes in viscosity are observed on binding to the DNA grooves.^52^ We found that (Fig. 5 and S8†) no change in viscosity was observed upon the addition of multiple concentrations of α-AA to hs-DNA, leading to a strong indication that α-AA binds to the minor groove of hs-DNA.

### Circular dichroism (CD) spectroscopy

**Fig. 6:**
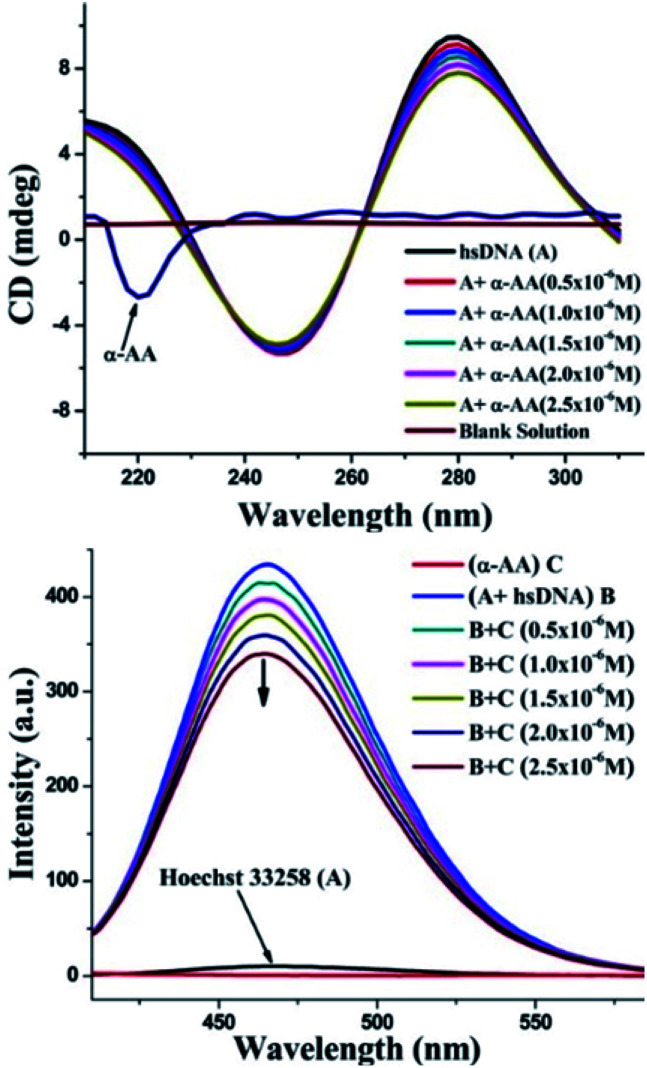
(Top) Effect of α-AA on CD spectra of hs-DNA. CD spectra of hs-DNA with varying concentration of α-AA (0.5–2.5 × 10^−6^ M). (Down) Fluorescence spectra of the Hoechst–hs-DNA complex with α-AA. Hoechst–hs-DNA complex emission intensities were recorded from 350–600 nm and excited at 343 nm.

As seen in Fig. 6, the CD spectrum of DNA reveals a base stacking +ve band at 275 nm and polynucleotide helicity −ve band at 245 nm.^53^ These two characteristic bands (275 nm and 245 nm) of DNA and interaction with molecules cause significant changes in the CD spectrum of the DNA system.^54,55^ Intercalative binding results in an increase in the intensity of the signal at 275 nm (stacking of the intercalator between DNA base pairs).^56^ In contrast, groove binding molecules do not cause significant change in the CD spectrum of DNA due to the retention of the DNA structure without significant unwinding of the DNA base pairs.^57^ In our experiments, a moderate decrease in the intensity of the positive band was seen, while there were only minor perturbations in the negative CD band without any blank interference (Fig. S9 and S10†). No new bands or change in the band shape was observed. Thus, there were no significant conformational changes/unwinding of DNA base pairs/changes in the form of DNA upon the addition of α-AA, indicating that α-AA binds in the groove region of DNA.^58^ This observation also corresponds with the DNA denaturing study that was discussed previously.^59^

### Minor groove displacement assay

To investigate the precise mode for the binding of α-AA to DNA, competitive binding experiments were performed with Hoechst 33258. Hoechst 33258 is a known DNA minor groove binder.^60^ The Hoechst dye shows diminished fluorescence in liquid solutions. However, in the existence of DNA, its emission is greatly increased.^61^ Any small molecule that interacts with DNA *via* a similar minor groove binding mechanism will displace the Hoechst from the groove, and hence, cause the quenching of the fluorescence intensity of the system.^52^ In our experiments, the addition of α-AA caused a significant decrease in the intensity of the Hoechst–DNA system (Fig. 6). The possibility of α-AA interacting with Hoechst was refuted based on the observation that the fluorescence intensity of Hoechst does not change appreciably upon the addition of α-AA (0.5–2.5 × 10^−6^ M) (Fig. S11†), indicating that α-AA does not interact with Hoechst. Hence, the decrease in the fluorescence intensity of the hs-DNA–Hoechst system indicates the displacement of bound Hoechst competing for the same site on the hs-DNA (minor groove).^60^

### *In silico* molecular docking

**Fig. 7:**
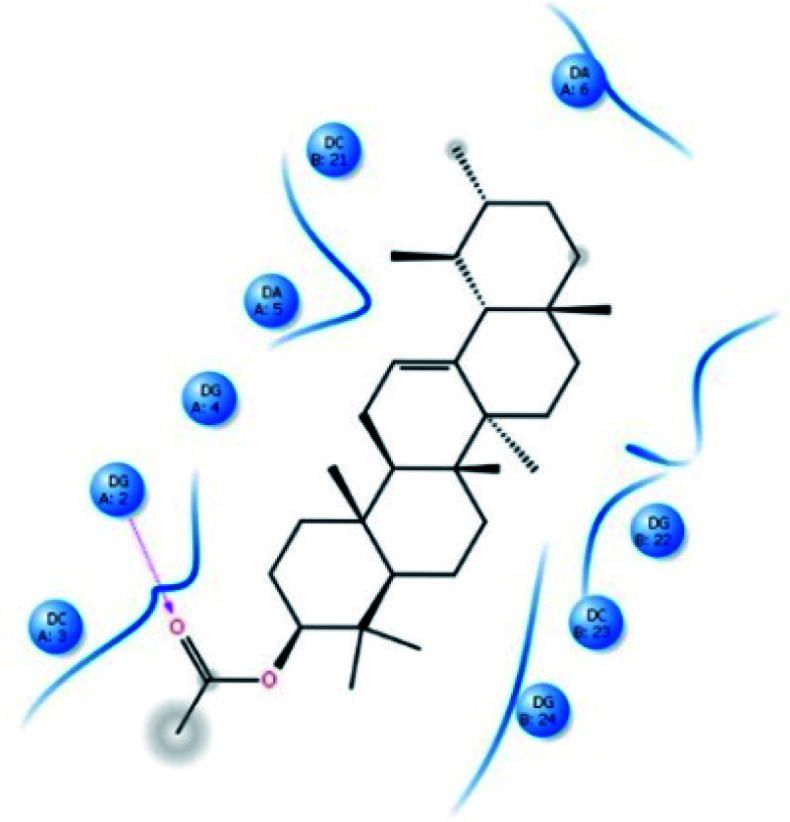
Schematic representation of the binding mode between α-AA and 1BNA. The red-dashed line shows the hydrogen bond interactions.

α-AA was successively docked with DNA dodecamer d(CGCGAATTCGCG)_2_ (PDB ID: 1BNA) to predict the site and binding energy between α-AA and DNA.^62^ The energetically most favourable conformation of the docked pose (Fig. 7 and S12†) indicated that α-AA interacts with the DNA minor groove site. It was located within the adenine(A)–thymine (T) (10.8 A°) region in comparison to the guanine (G)–cytosine (C) (13.2 A°) region as per the Hoechst 33258 (A–T rich binder) replacement experiment involving hydrogen bonding and van-der-Waals interaction with DNA functional groups. Similar observations were made earlier in the case of minor groove binders.^63^

From the docking calculations, the free energy change (Δ*G*) for the DNA–α-AA complex was found to be −26.85 kJ mol^−1^, which is comparable to the experimentally obtained free energy of binding (−28.41 kJ mol^−1^ from fluorescence quenching experiments).

## Conclusions

This study deals with important findings of biophysical sensing studies between hs-DNA and a biomimetic natural product α-AA. The binding between hs-DNA and small molecule α-AA has been deciphered using a multitude of complementary techniques. Spectrophotometric analysis indicates the formation of a minor groove–bound complex between α-AA and hs-DNA. Observed thermodynamic energies indicate that the binding process is spontaneous due to hydrophobic sensing behavior. Thus, it is conceivable that the α-AA–hs-DNA biological sensing probe acts at the edge of the chemical–biology interface. In conclusion, we have used a combination of biophysical techniques to obtain valuable information on the mechanism of binding of small molecule α-AA to hs-DNA *in vitro* used informatively for directed evolution between them.

## Conflicts of interest

The authors declare no competing financial interests.

## Supplementary Material

RA-012-D1RA07195E-s001
