# Supplementary material for: Deciphering the sensing of α-amyrin acetate with hs-DNA: a multipronged biological probe
Source: RSC Adv. 2022 Jan 5;12(3):1238–43. doi: 10.1039/d1ra07195e (PMC8978960; doi:10.1039/d1ra07195e)
Supplement: RA-012-D1RA07195E-s001 [file RA-012-D1RA07195E-s001.pdf]

Electronic Supplementary Material (ESI) for RSC Advances.
This journal is © The Royal Society of Chemistry 2021

Supporting Information

Deciphering the interaction of α-Amyrin Acetate with hs-DNA: A multipronged biological probe

Amol V. Pansare,[a,b*‡] Amol A. Shedge,[b‡] Maryappa C. Sonawale,[c] Shubham V. Pansare,[b] Akshay D. Mahakal,[b] Shyam R. Khairkar,[b] Shraddha Y. Chhatre,[d] Dnyaneshwar K. Kulal,[b] Vishwanath R. Patil[b*]

[a]Composite group, Engineering, Swiss Federal Laboratories for Materials Science and Technology-Empa, 8600 Dübendorf, Switzerland. E-mail:amol.pansare@empa.ch

[b]Department of Chemistry, University of Mumbai, Santacruz (E), Mumbai 400098, India. E-mail: vishwanathrpatil03@gmail.com

[c]Veer Wajekar Arts Science and Commerce College, Navi Mumbai 400702, India.

[d]National Chemical Laboratory (NCL) Dr. Homi Bhabha Road, Pune 411008, India.

Isolation and characterizations of α-AA

α-AA was isolated from ethanol extract of *F. arnottiana* leaves using silica gel open column chromatography. First mobile phase (hexane), 2nd mobile phase (hexane and chloroform in 1:1 ratio), 3rd mobile phase (Chloroform), yellow colored broad band fraction separated from ethanol leaves extract. Evaporated yellow colored fraction up to dryness, the yellowish white colored compound were obtained, which were again treated with silica gel and aluminum oxide active neutral (1:1ratio) pencil column using chloroform and n-hexane (1:5 ratio) as mobile phase, the white colored compound have been separated.

IR spectum was recorded on Perkin Elmer FT-IR spectrometer Frontier using ATR; transmittances at 4000 to 400 cm^{-1}. The Bruker AMX-300 MHz NMR spectrometer (France) was used to record ^{1}H NMR and ^{13}C NMR data. **^{1}HNMR(300MHz, $CDCl_3$):** δ 0.96-1.01(m, 23H), 1.06(s, 3H), 1.31(s, 3H), 1.25-1.41(m, 14H), 1.37(s, 3H), 1.41(s, 3H), 1.51(s, 3H), 1.89-1.93(m, 4H), 2.04(s, 3H), 4.48(t, 1H), 5.11-5.19(m, 1H). **^{13}C NMR (75.46 MHz, $CDCl_3$):** δ 21.30, 23.23, 23.37, 23.61, 28.07, 31.25, 32.87, 33.75, 36.80, 37.71, 38.47, 39.65, 39.65, 40.03, 41.54, 42.07, 47.65, 55.27, 59.07, 80.94, 124.32, 139.63, 170.98.

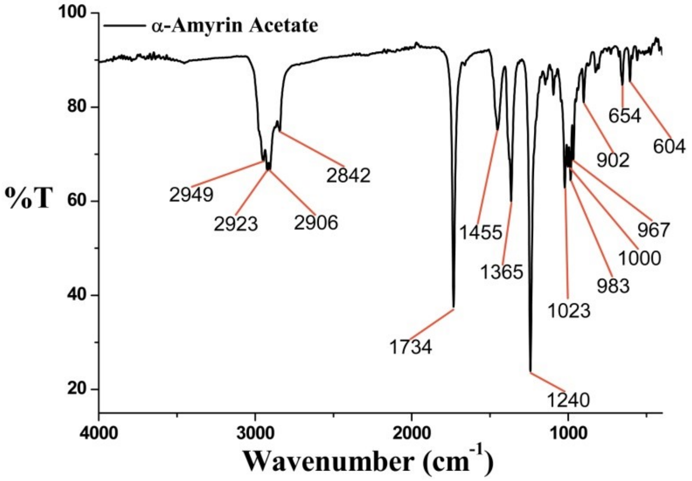

Figure S1. FT-IR spectrum of α-AA, which showed transmittance bands at 2949, 2923, 2906 and 2842 cm^{-1} indicated the presence of an aliphatic alkyl moiety while the other bands were assigned for 1734 (C=O), 1455 (C=C); 1365 (CH3- / CH3CO), 1240 (−C-O) and 1023, 1000, 983, 967, 902, 654, 604 cm^{-1} (C-H).)

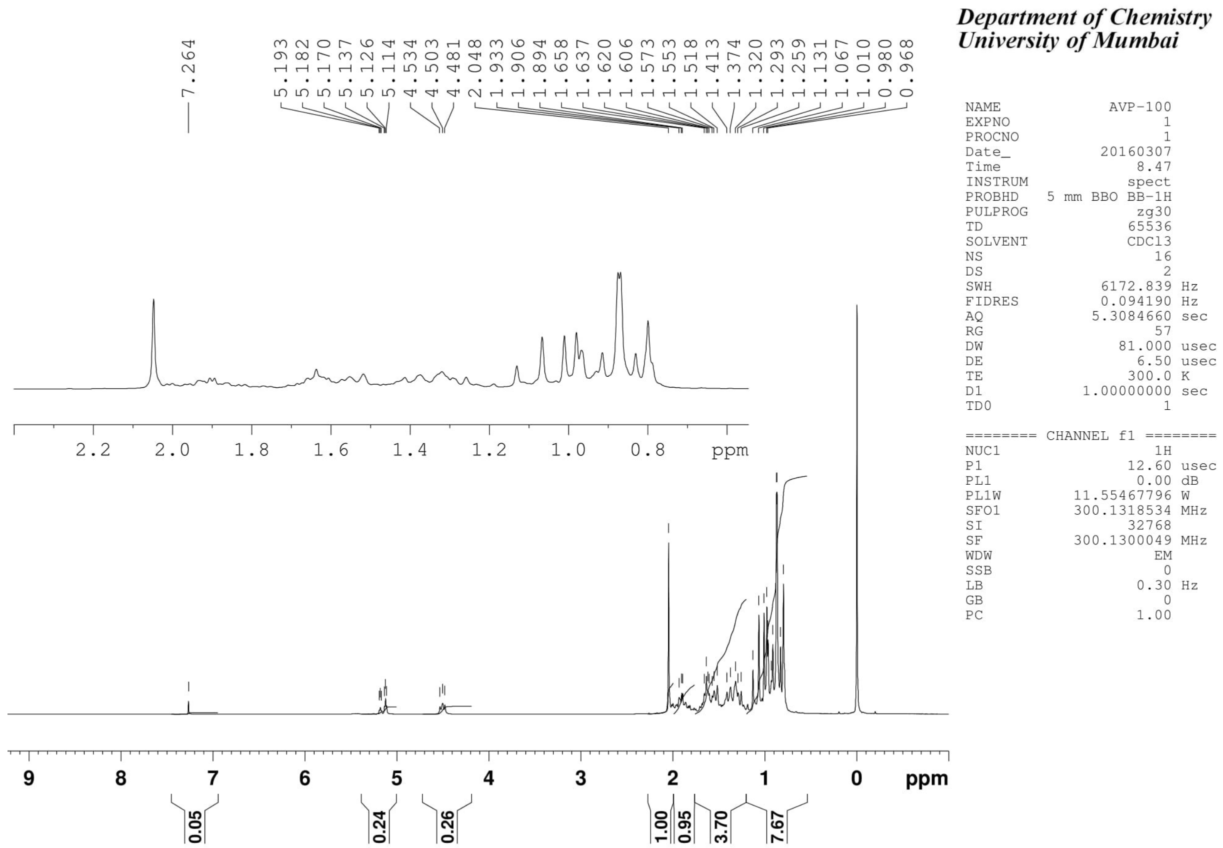

Figure S2. ^{1}H NMR of α-AA, was the complex spectrum. Intriguingly ^{1}H NMR consist of six singlet for seven methyl protons appeared at δ1.31, 1.25, 1.37, 1.41, 1.51, 2.04 ppm respectively. One of the methyl singlets, which appeared downfield at δ 2.02 ppm, was indicative of the presence of acetate group. Furthermore, the triplet resonated at δ 4.48 ppm confirmed the signal for the proton directly attached to the ester oxygen. Moreover, the multiplet appeared at δ 5.11 to 5.19 ppm, assigned for one of the olefinic proton. However, the NMR showing the other additional complex nonresolvable multilets at δ 0.96-1.01, 1.25-1.41 and 1.89-1.93 ppm observed for the coupling among the methyl, methylene and methyne protons of α-AA compound.

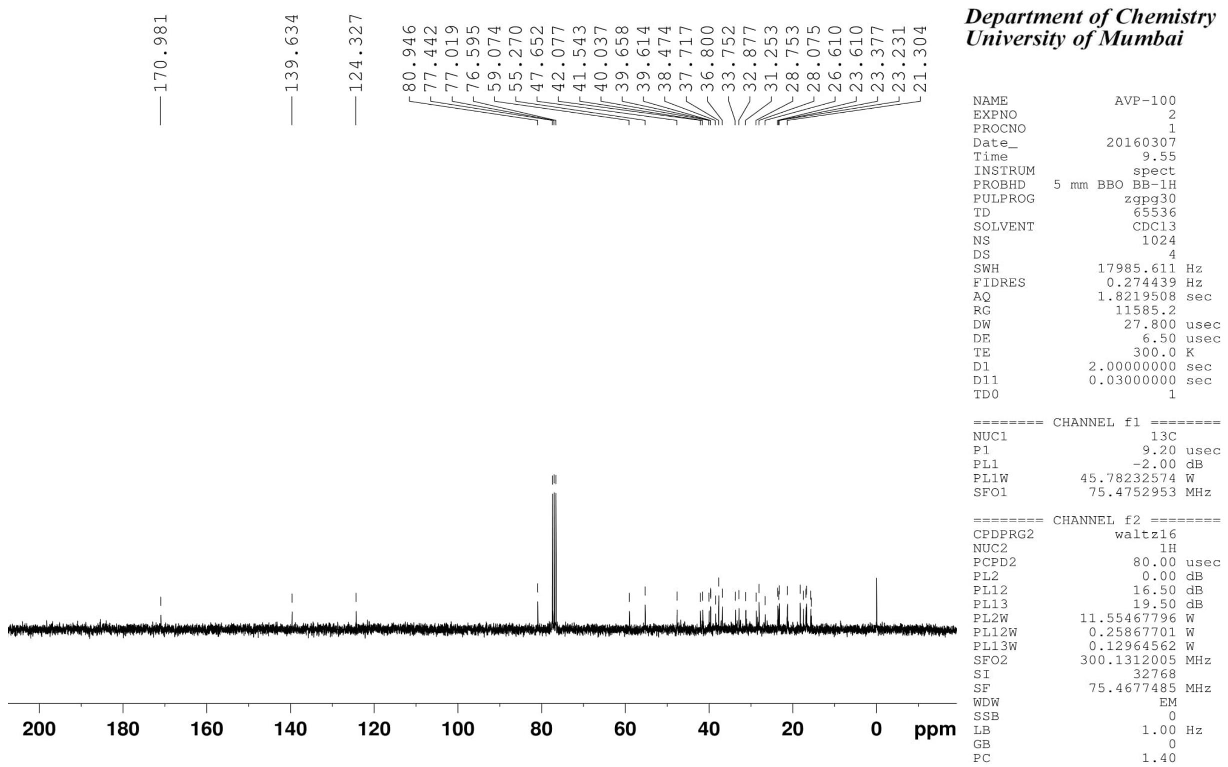

Figure S3. ^{13}C NMR spectrum of α-AA, observed the few prominent signals out of which, signal appeared at δ 80.94 ppm assigned for carbon directly attached to oxygen of the acetate group. Further, the signal at δ124.32 and 139.63 ppm assigned for the two alkene carbons. The signal resonated at δ170.98 ppm confirmed the presence of acetate carbonyl group. However, the other carbon signals for α-AA compound, appeared in the range of δ 21.30 to 59.07 ppm and few carbon signals overlap with other carbon signals that results the less number of the carbon signals observed in the ^{13}C NMR.

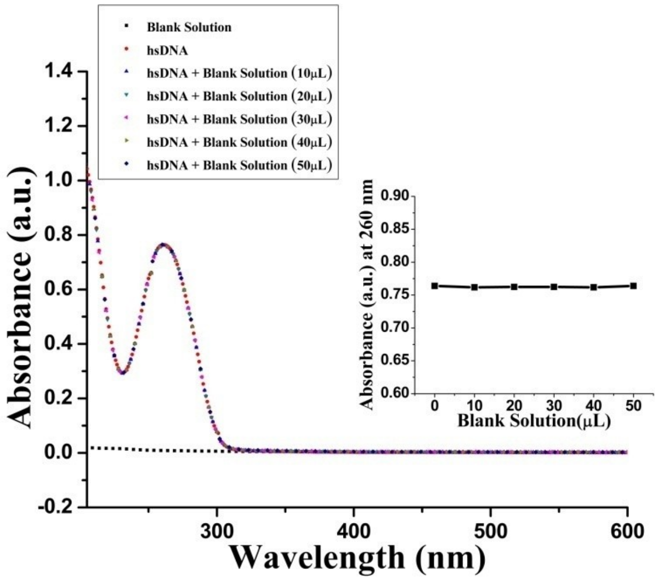

Figure S4. UV-Vis spectra of DNA at increasing concentration of blank solution (5% ethanol in Tris-HCl buffer solution) 10μl to 50μl, no any shift occurred during blank titration study.

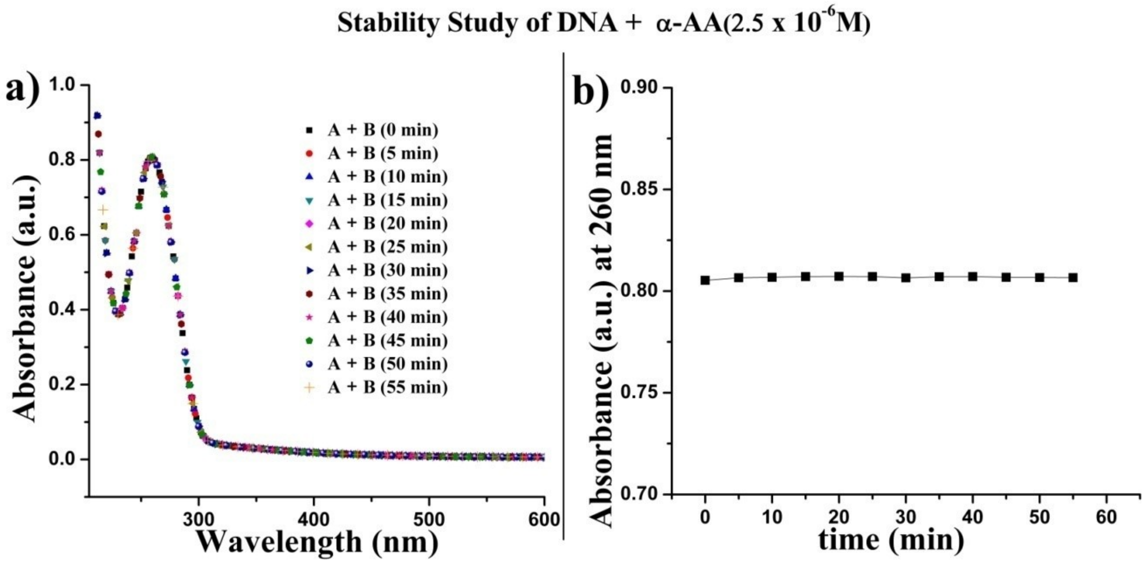

Figure S5. UV-Vis spectra of hsDNA(A) + α-AA(B) ($2.5x10^{-6}$M) at 260 nm for incresing time interval. Spectra suggested that no effect occurred in absorbance at 260nm during increasing time interval at highest concentration of α-AA, hence stability study of DNA-α-AA complex was obeyed.

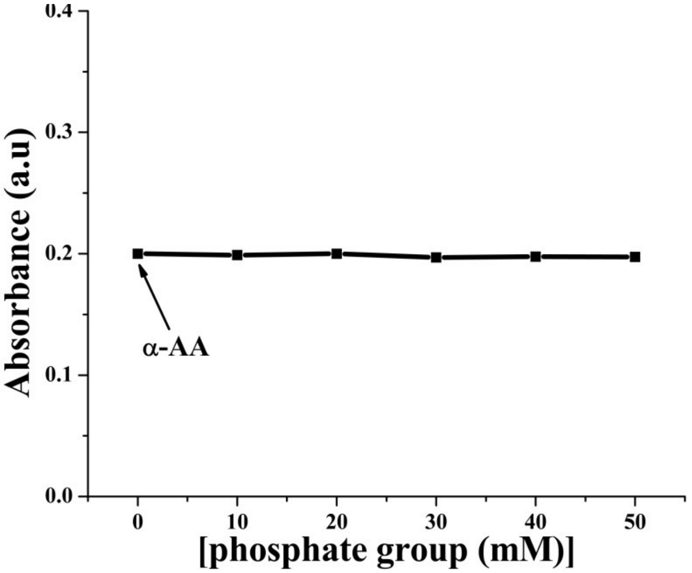

Figure S6. UV-visible absorption for α-AA with increasing phosphate group concentration, [α-AA] = 2.5 x10^{-6} M.

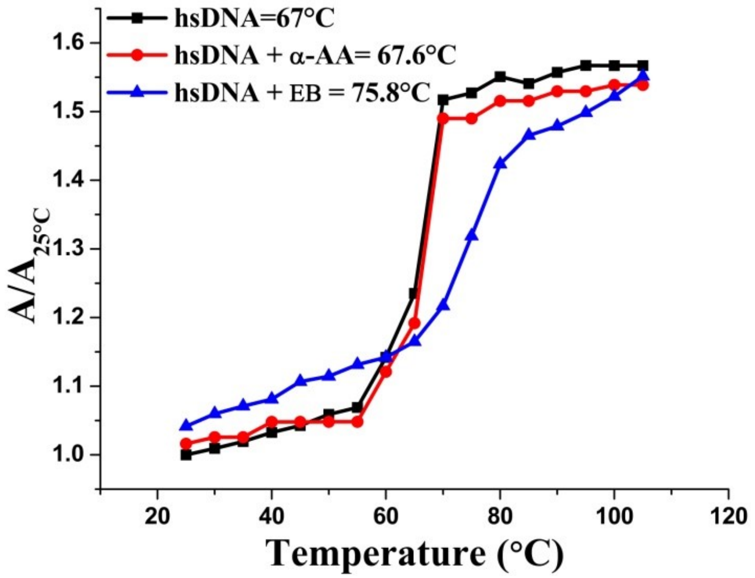

Figure S7. Melting transition analysis of hs DNA in the presence of α-AA and EB.

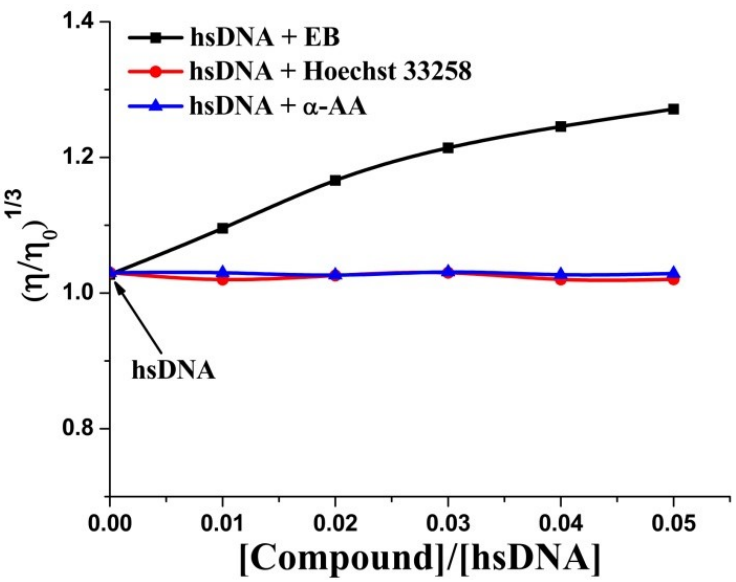

Figure S8. Effect of increasing amounts of α-AA, Hoechst 33258 and EB on the viscosity of hsDNA ($50x10^{-6}$M) respectively in Tris HCl buffer at 298 K [Compound]/[DNA]. A constant concentration of hs-DNA ($50\ x10^{-6}$M) was maintained.

The viscosity of three solutions were measured: (1) the viscosity in absence and presence of various concentrations of α-AA ($0.5\text{-}2.5x10^{-6}$M) (2) the viscosity in absence and presence of various concentrations of Hoechst 33258 ($0.5\text{-}2.5x10^{-6}$M) and (3) viscosity in absence and presence of various concentrations of EB respectively ($0.5\text{-}2.5x10^{-6}$M). The results of the viscosity experiments confirm that α-AA do not cause significant increase to the hs-DNA solution viscosity compared to the well established intercalator EB. Hoechst 33258 reagent is used as a positive control to represent a minor groove binder. The viscosity readings for the Hoechst 33258 compound were similar to that of α-AA.

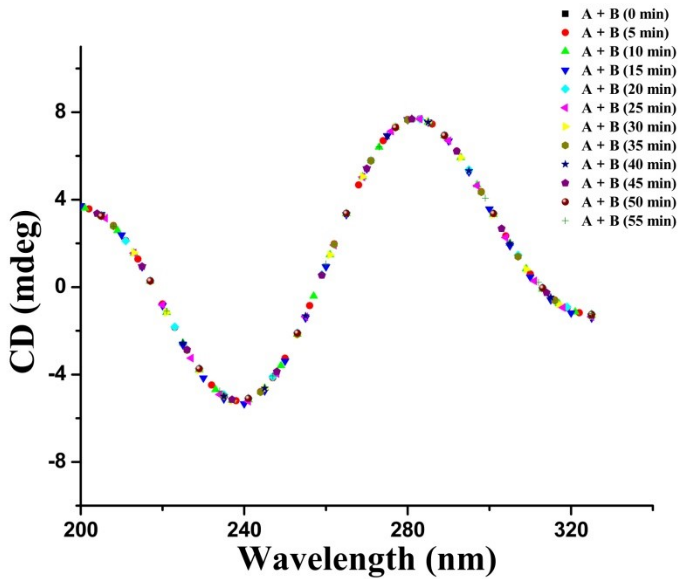

Figure S9. CD spectra of hsDNA(A) + α-AA(B) (2.5x10^{-6}M) at increasing time intervals (0 to 55 min). Spectra suggest that no change occurrs during the experiment at the highest concentration of α-AA, confirming the stability of DNA-α-AA complex over the time of the experiment in the buffer system.

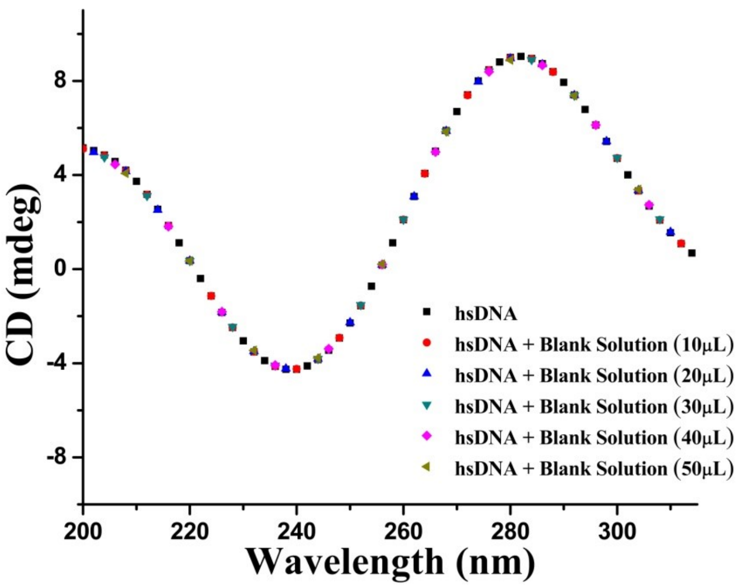

Figure S10. CD spectra of DNA at increasing concentrations of blank solution (5% ethanol in Tris-HCl buffer solution, 10µl to 50µl). No shift occurred during blank titration study.

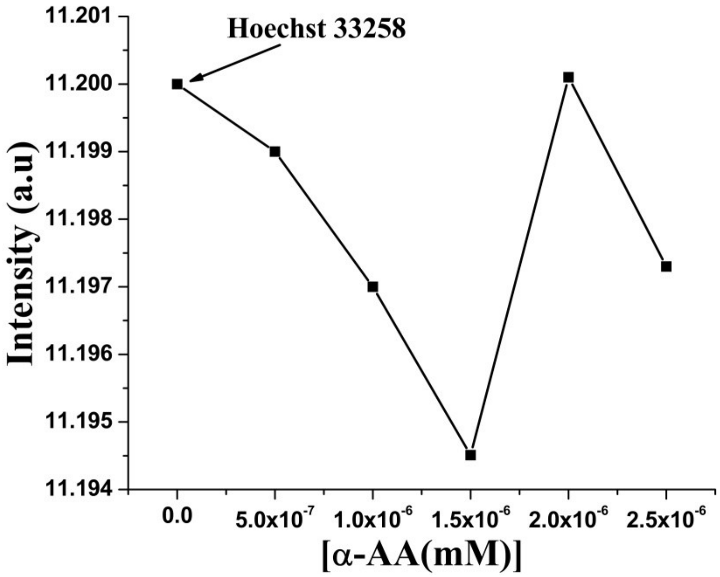

Figure S11. Fluorescence intensity of Hoechst 33258 with varying concentrations of α-AA (0.5-2.5x10^{-6}M).

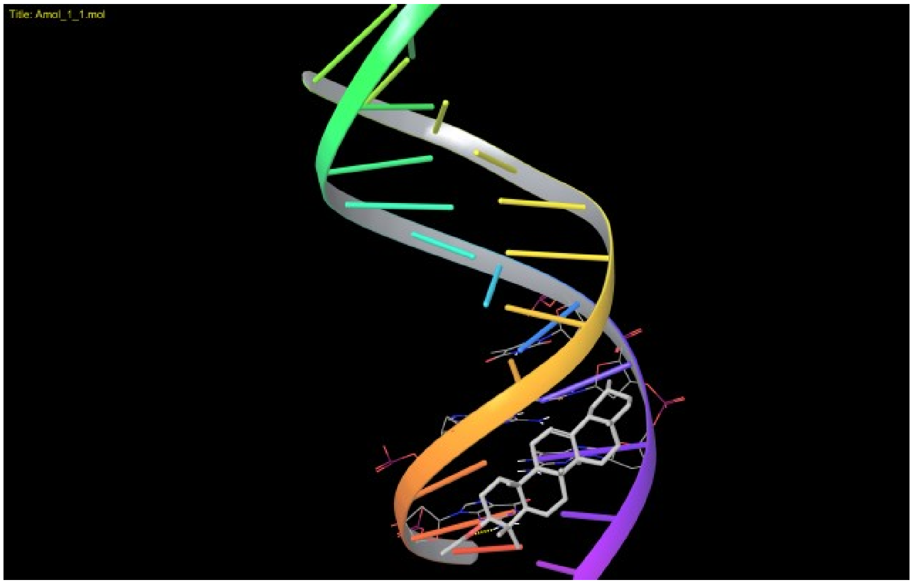

Figure S12. Molecular docked structure of DNA-α-AA complex. Dodecamer duplex sequence $(CGCGAATTCGCG)_2$ (PDB ID: 1BNA) was used in the docking studies. Full view of docking between -α-AA and 1BNA

Eqn S1. Sterm-Volmer equation

$$F_0/F = 1+ Ksv\,[Q] = 1+ Kq.\tau_0\,[Q]$$

Where, F_0 and F denotes the fluorescence intensities of hs DNA in absence and presence of α-AA, *Ksv* is the Stern-Volmer quenching constant, [Q] is the concentration of the α-AA, *Kq* is the biomolecular quenching rate constant, τ_0 is the average lifetime of the molecule without the quencher which is equal to 10^{-8} s.

Eqn S2. Double logarithmic Stern-Volmer equation .

$$\text{Log}\,[(F_0\text{-}F)/F] = \text{Log}K + n\log[Q]$$

Where, *K* is binding constant and *n* is the number of binding sites.

Eqn S3. Vant Hoff's equation

$$\ln K = (-\Delta H^0/RT) + (\Delta S^0/R)$$

Where, *K*, *R* and *T* are binding constant, gas constant and temperature respectively.

Eqn S4. Gibbs free energy (ΔG^0)

$$\Delta G^0 = \Delta H^0 - T\Delta S^0 = -RT\ln K$$

Where, ΔS^0 is change in entropy and ΔH^0 is enthalpy.

Table S1. Binding parameters of green synthesized α-AA with hsDNA at various temperatures.

**Temperature (K)**	***Ksv*** **(M^{-1})**	***Kq*** **($M^{-1}s^{-1}$)**	***K*** **(LM^{-1})**	***n***
293	7.05×10^{4}	7.05×10^{12}	7.75×10^{4}	1.0056
298	5.58×10^{4}	5.58×10^{12}	3.52×10^{4}	0.9634
310	4.70×10^{4}	4.70×10^{12}	2.08×10^{4}	1.2921

Table S2: Thermodynamic parameters of α-AA -hsDNA interaction.

**Temperature (K)**	**ΔH^0 ($KJmol^{-1}$)**	**ΔS^0 ($Jmol^{-1}K^{-1}$)**	**ΔG^0 ($KJmol^{-1}$)**
293			-25.74
298	130.48	533.23	-28.41
310			-34.81
